# Risk factors for neurosyphilis in HIV patients: A retrospective cohort study

**DOI:** 10.1016/j.bjid.2025.104519

**Published:** 2025-03-28

**Authors:** Beatriz Arns, Tarsila Vieceli, Eduardo Gomes, Mariana Horn Scherer, Luisa Nakashima, Maria Luisa Santos, Ronara Blos Hepp, Fernanda Greinert, Maria Helena Rigatto

**Affiliations:** a Hospital de Clínicas de Porto Alegre, Programa de Pós-Graduação em Ciências Médicas, Porto Alegre, RS, Brazil; b Universidade Federal do Rio Grande do Sul, Porto Alegre, RS, Brazil; c Pontifícia Universidade Católica do Rio Grande do Sul, Porto Alegre, Brazil; d Universidade Federal do Rio Grande do Sul, Departamento de Medicina Interna, Porto Alegre, RS, Brazil

**Keywords:** HIV, Syphilis, Neurosyphilis, Lumbar puncture, Risk factors

## Abstract

**Introduction:**

Syphilis is a highly prevalent sexually transmitted infection worldwide. Patients living with Human Immunodeficiency Virus (HIV) have a higher risk of developing neurosyphilis. Actual guidelines advise to proceed with lumbar puncture only if neurologic symptoms are present. However, asymptomatic neurosyphilis patients are not rare in the HIV population and other risk factors should be defined to guide screening.

**Methods:**

We performed a retrospective cohort to evaluate risk factors related to neurosyphilis in HIV patients. Adults with HIV infection and laboratory confirmed syphilis between 2011 and 2021 were included. Patients with no record of syphilis treatment, VDRL titers ≤ 1:4, other neurologic diseases or non-HIV related immunological impairment were excluded. The patients were followed for 2-years after syphilis diagnosis.

**Results:**

One-hundred and forty patients (190 syphilis episodes) were included, with mean age of 45.0 ± 9.2-years-old, 111 (79.3 %) were male, 48 (25.8 %) had CD4 count ≤ 350 cells/mm^3^ at syphilis diagnosis (median: 522.5 cells/mm^3^; IQR: 315.5‒703.5), 127 (66.8 %) of 172 had a HIV viral load ≤ 400 copies/mm^3^ and median serum VDRL titer was 1:64 (IQR: 1:16‒1:128). In multivariate analysis, serum VDRL titers ≥ 1:32 and the presence of neurologic symptoms were associated with neurosyphilis, while HIV viral load ≤ 400 copies/mm^3^ was a protective factor.

**Discussion:**

In addition to the presence of neurological symptoms, HIV viral load > 400 copies/mm^3^ and VDRL titers ≥ 1:32 were shown to be risk factors for neurosyphilis in this study and diagnostic lumbar puncture should be considered in these cases.

## Introduction

Syphilis is a systemic bacterial infection caused by *Treponema pallidum*, a Gram-negative bacterium from the spirochete group. It is transmitted sexually or vertically and there is worldwide concern due to the progressively increasing incidence that has been observed in recent years.^1^^,^^2^ Neurosyphilis is usually a late complication of untreated syphilis which occurs in approximately 30 % of the patients^3^ Syphilis incidence is estimated to be 77-times greater in HIV infected individuals than that of the general population.^4^^,^^5^ Untreated syphilis facilitates HIV transmission; on the other hand, HIV infection interferes with the clinical manifestations of syphilis and increases risk of treatment failure.^6^ Additionally, due to immunosuppression, progression to neurosyphilis is more common among HIV patients, in which about 30 % of patients with untreated syphilis develop neurosyphilis.^7^

The benefit of CSF analysis in HIV patients with syphilis and neurological symptoms is clear. The Centers for Disease Control (CDC) 2013 guidelines proposed that only patients with clinical evidence of neurological involvement ‒ such as cognitive dysfunction, motor or sensory deficit, visual or hearing symptoms, among others ‒ should be submitted to Cerebrospinal Fluid (CSF) analysis.^8^ However, diagnosing neurosyphilis in asymptomatic patients poses a challenge. A retrospective cohort study evaluated 7083 patients with syphilis. This cohort showed that the incidence of neurosyphilis among those infected with HIV compared to those without HIV was 2.1 % and 0.6 %, respectively, culminating in 67 % of neurosyphilis cases was diagnosed in HIV-infected participants (21 % were asymptomatic).^9^

Since Central Nervous System (CNS) involvement can occur at any syphilis stage,^10^ and CSF abnormalities are common in patients with early syphilis,^11^^,^^12^ even in the absence of symptoms, submitting only symptomatic patients to CSF analysis could lead to underdiagnosis of neurosyphilis. Some studies reported a greater level of neurocognitive impairment or CNS inflammation in HIV positive patients with previous early-syphilis but no diagnosis of neurosyphilis who were treated with standard benzathine penicillin G, which does not cross the blood-brain barrier.^13^^,^^14^ However, it is often not possible to submit all patients with HIV infection and syphilis to a lumbar puncture. Potential risk factors other than neurological symptoms could help base the decision on which patients should undertake CSF analysis, such as viral load, CD4 count and VDRL titers. Therefore, this study aims to evaluate the impact of these factors on the development of neurosyphilis in a two-year span in a high-HIV prevalence setting.

## Materials and methods

### Study design, patients and settings

We performed a retrospective cohort of adult patients (> 18-years-old) with HIV infection who were diagnosed with syphilis between 2011 and 2021 and were followed in outpatient clinic or in-hospital care in two teaching hospitals (335 and 836 beds each) in the city of Porto Alegre, Brazil.

Patients were excluded if there was no record of syphilis treatment or if other neurologic diseases or non-HIV related immunological impairment were present. Patients presenting VDRL titers ≤ 1:4 were also excluded as we could not rule out the possibility of a false-positive result in previously treated patients. Patients were followed for two years after syphilis diagnosis.

### Variables and definitions

Syphilis diagnosis was made by a positive treponemal titer (FTAbs) followed by a positive non-treponemal test (VDRL). In previously diagnosed patients, a new syphilis diagnosis was considered when VDRL titer raised ≥ 4-fold or 2 dilutions. Neurosyphilis was defined as syphilis diagnosis and CSF with positive VDRL or leukocyte count > 10 cells/mm^3^. As HIV itself can cause CSF pleocytosis and the threshold for neurosyphilis diagnosis is not a consensus, we also explored how diagnostic rates would be impacted if we considered a CSF leukocyte count criteria of ≥ 20 cells/mm^3^. Adequate response for latent syphilis treatment was defined as serological response (defined as a ≥ 4-fold or 2 dilutions decrease in VDRL titer or reversion of the test to nonreactive) 1 year after treatment in the absence of neurological symptoms within 2 years. Reinfection was defined as ≥ 4-fold or 2 dilutions increase in VDRL titers during follow-up.

The primary outcome was confirmed neurosyphilis within 2 years of follow-up. Follow-up losses were considered as not having the primary outcome, favoring the null hypothesis.

Potential risk factors for neurosyphilis development evaluated were: demographic characteristics (age and sex); clinical neurological symptoms (headache, dizziness, stroke, meningism, coma, seizures, gummas, visual and otologic symptoms); antiretroviral therapy; antimicrobial treatment for syphilis and laboratory parameters (VDRL, FTAbs, CSF biochemistry, CD4+ lymphocyte, HIV viral load). Data were collected through review of medical records. Both centers have neurologists on-site for medical care, but patients were only evaluated in cases that the attending team requested based on patient complaints.

### Statistical analysis

The data were presented as median and Interquartile Range (IQR), 25th (p25) and 75th (p75) percentiles, for ordinal or non-normally distributed continuous variables and total and percentage values for categorical variables. Bivariate analysis was performed separately for each variable to evaluate the differences between patient outcomes groups (confirmed neurosyphilis versus others); p-values were calculated using Fisher's exact test for categorical variables and Student's *t*-test or Wilcoxon-Mann-Whitney test for continuous variables. We also explored whether the change in the 2015 CDC guideline recommendation for performing lumbar puncture only in symptomatic patients impacted clinical practice by comparing patients’ profiles and diagnosis in the two periods.

A logistic regression model was set to evaluate risk factors for developing neurosyphilis. Variables with p < 0.2 in univariable analysis were included in the model, and variables with p < 0.05 in the multivariate analysis remained in the final model. We performed an alternative analysis considering only patients with CSF positive VDRL titers as having confirmed neurosyphilis. Subgroup analysis was performed: 1) Excluding patients that did not reach serologic response and were not submitted to lumbar puncture, as we could not rule out neurosyphilis; 2) Including only patients who were submitted to CSF analysis; and 3) Excluding patients who presented with neurological symptoms. All tests were two-tailed and a p ≤ 0.05 were considered significant. Analyses were performed in SPSS, version 29.0.

### Ethical aspects

This study was approved by the ethics committee of the involved hospitals (CAAE 89054518.5.0000.5336 and 89054518.5.3001.5327). The informed consent was waived by the ethics committee due to retrospective data collection from medical records, without direct interventions to participants.

## Results

### Cohort characteristics

**Fig. 1:**
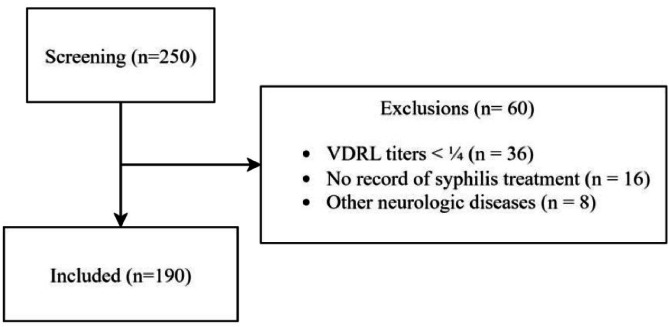
Inclusion flowchart.

From 2011 to 2021, 250 registered syphilis episodes from patients followed up in the HIV outpatient clinic were evaluated for this cohort (Fig. 1). From these, 36 (14.4 %) were excluded for VDRL titers < 1:4 at diagnosis, 16 (6.4 %) for no registry of treatment and 8 (3.2 %) for other neurologic conditions. One-hundred and ninety syphilis episodes were included for analysis, accounting for 140 patients. Patients' median age was 46.4 (34.0‒54.0) years old and 111 (79.3 %) were male. Forty-nine (25.8 %) episodes occurred in patients who had CD4 count < 350 cells/mm^3^ at syphilis diagnosis. Median CD4 count was 522.5 cells/mm^3^ (IQR: 315.5‒703.5). One-hundred and twenty-seven (66.8 %) of 172 had a HIV viral load < 400 copies/mm^3^. Median VDRL titer was 1/64 (IQR: 1/16‒1/128). To treat these syphilis episodes, the patients received penicillin benzathine 147 (77.4 %), penicillin cristalin 17 (8.4 %), ceftriaxone 27 (14.2 %), doxycycline 14 (7.4 %).

In 84 (44.2 %) of the 190 syphilis episodes, patients were submitted to lumbar puncture. Twenty-five (29.8 %) of these patients had a confirmed diagnosis of neurosyphilis: 19 (76.0 %) of 25 due to positive CSF VDRL titers and 6 (24.0 %) due to cytologic criteria with median leukocyte count of 16 (IQR: 15‒80) cells/mm^3^. Four of the six patients who were diagnosed due to cytologic criteria had CSF leukocyte count < 20 (12‒16) cells/mm^3^. Therefore, if a threshold of ≥ 20 cells/mm^3^ was considered for diagnosis, 21 (25.0 %) of 84 patients who performed lumbar puncture would meet the criteria for confirmed neurosyphilis.

Among the 165 latent syphilis episodes, 142 (86.1 %) fulfilled cure criteria after treatment (twofold reduction in subsequent serum VDRL test titers and no clinical symptoms in two years). In the 23 (13.9 %) episodes that did not achieve cure criteria, patients had been treated with benzathine penicillin 19 (82.7 %), ceftriaxone 2 (8.7 %), doxycycline 2 (8.7 %) and cristalin penicillin 1 (4.3 %).

When comparing patients who performed lumbar puncture from 2011‒2014 and 2015‒2021, 4 (8.2 %) of 49 had neurologic symptoms versus 12 (34.8 %) of 35, respectively, p = 0.004. Confirmed neurosyphilis rates were 11 (12.8 %) of 86 and 14 (13.5 %) of 104, in the first and second period of the cohort respectively, p = 0.99. Nine (81.8 %) of 11 patients diagnosed with neurosyphilis were asymptomatic between 2011‒2014, versus 7 (50.0 %) of 14 patients in the second period, p = 0.21.

### Risk factors for neurosyphilis

Risk factors for developing neurosyphilis in univariate analysis were presented in Table 1. In the multivariate logistic regression model, neurologic symptoms Odds Ratio (OR = 4.23), 95 % Confidence Interval (95 % CI 1.50‒11.93), p < 0.01, HIV viral load ≤ 400 copies/mm^3^ (OR = 0.28, 95 % CI 0.11‒0.70, p < 0.01) and VDRL serological titers ≥ 1:32 (OR = 5.1, 95 % CI 1.11‒23.0, p = 0.036) were independently related factors. In an alternative analysis, considering only CSF positive VDRL titers as confirmed neurosyphilis criteria, neurologic symptoms (OR = 5.74, 95 % CI 1.72‒19.09, p = 0.004), VDRL serological titers ≥ 1:32 (OR = 8.9, 95 % CI 1.10–73.4, p = 0.043) and CD4 count ≤ 350 cells/mm^3^ (OR = 4.4, 95 % CI 1.5‒13.6, p < 0.001) were independent risk factors, while HIV viral load ≤ 400 copies/mm^3^ (OR = 0.25, 95 % CI 0.8‒0.76, p = 0.015) was a protective factor.

**Table 1:** Risk factors for developing neurosyphilis in univariate analysis.

Risk factors	Total Cohort	Neurosyphilis		
	n = 190	Yes = 24	No = 166	p
Age (years)	46.4 (34.0‒54.0)	49.0 (41.5‒53.3)	44.2 (34.0‒54.0)	0.318
Gender (male)	148 (77.9)	10 (80.0)	128 (77.6)	0.99
CD4+ < 350 cells/mm^3^	49 (25.8)	11 (44.0)	38 (23.0)	0.047
VDRL titer > 1:32	133 (70.0)	23 (92.0)	110 (66.7)	0.009
HIV viral load < 400 U/mm^3^	127 (66.8)	10 (40.0)	117 (70.9)	0.005
Neurologic symptoms*	24 (12.6)	9 (36.0)	15 (9.1)	0.001
Headache	8 (4.2)	0	8 (4.8)	0.600
Dizziness	5 (2.6)	3 (12.0)	2 (1.2)	0.017
Stroke	6 (3.2)	4 (16.0)	2 (1.2)	0.003
Meningism	1 (0.5)	0	1 (0.6)	0.99
Sensorium depression	3 (1.6)	2 (8.0)	1 (0.6)	0.046
Seizures	1 (0.5)	1 (4.0)	0	0.132
Panuveitis	2 (1.1)	1(4.0)	1(0.6)	0.246
Venous sinus thrombosis	1 (0.5)	1(4.0)	0	0.132

### Subgroup analysis

We did subgroup analysis excluding patients initially considered to have latent syphilis but who failed to achieve cure criteria after treatment, considering that neurosyphilis could not be completely excluded in this population. One-hundred and sixty-seven syphilis episodes were included in this model. HIV viral load ≤ 400 copies/mm^3^ was an independent protective factor (OR = 0.29, 95 % CI 0.15‒0.72, p = 0.007), while neurological symptoms increased the risk for neurosyphilis diagnosis (OR = 4.6, 95 % CI 1.64‒12.69, p = 0.004).

We also evaluated separately only the subgroup of patients who performed lumbar puncture. Eighty-four patients were included in this analysis. Neurological symptoms (OR = 3.6‒4.5, 95 % CI 1.05‒12.1, p = 0.041), HIV viral load ≤ 400 copies/mm^3^ (OR = 0.28, 95 % CI 0.10‒0.81, p = 0.018) and VDRL serological titers ≥ 1:32 (OR = 5.5, 95 % CI 1.10‒27.7, p = 0.041) were the variables in the final model.

**Fig. 2:**
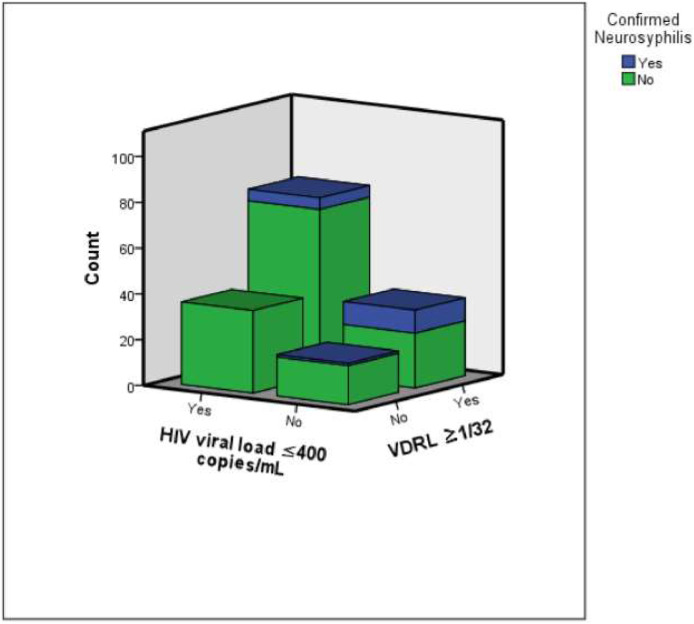
The figure illustrates the distribution of confirmed neurosyphilis cases among asymptomatic HIV-syphilis co-infected patients, stratified by VDRL serum titers (≥ 1/32 vs. < 1/32) and HIV viral load (≤ 400 copies/mL). Notably, no neurosyphilis cases were observed in patients with VDRL serum titers < 1/32 and HIV viral load ≤ 400 copies/mL.

Finally, we excluded patients who presented with neurological symptoms at syphilis diagnosis, in order to understand risk factors in the population of asymptomatic patients. One-hundred and sixty-six patients were included in this analysis, with 16 (9.6 %) neurosyphilis confirmed diagnosis: 11 with CSF VDRL positive titers and 5 due to cytologic criteria. An HIV viral load ≤ 400 copies/mm^3^ (OR = 0.20, 95 % CI 0.06‒0.65, p = 0.008) was an independently protective factor, while serum VDRL titer ≥ 1:32 (OR = 13.4, 95 % CI 1.6‒113.4, p = 0.017) and CD4 count ≤ 350 cells/mm^3^ (OR = 4.0, 95 % CI 1.23‒1289, p = 0.021) increased the risk for confirmed neurosyphilis. Considering only the 11 patients with CSF VDRL positive titers as having confirmed neurosyphilis, HIV viral load ≤ 400 copies/mm^3^ (OR = 0.16, 95 % CI 0.031‒0.83, p = 0.029) and CD4 count ≤ 350 cells/mm^3^ (OR = 10.0, 95 % CI 1.94‒51.21, p = 0.006) were independent risk factors to this outcome. Among 128 (77.1 %) of the 166 asymptomatic patients which had CD4+ ≤ 350 cells/mm^3^ or VDRL titer ≥ 1:32, 15 (11.7 %) had confirmed neurosyphilis: 11 with CSF VDRL positive titers and 4 due to cytologic criteria. In contrast, none of the asymptomatic patients with HIV viral load ≤ 400 copies/mm^3^ and VDRL titers ≤ 1:32 were diagnosed with neurosyphilis, see Fig. 2.

## Discussion

In this cohort of 190 syphilis episodes in HIV-infected patients, 25 (13.2 %) were diagnosed with neurosyphilis. Neurological symptoms and VDRL titers ≥1:32 were independent risk factors for confirmed neurosyphilis, while HIV viral load ≤400 copies/mm^3^ was a protective factor. The findings were the same in the subgroup in which all patients had CSF evaluation. When we considered only patients with CSF VDRL positive titers as having confirmed neurosyphilis, results were maintained and CD4 count ≤ 350 cells/mm^3^also appeared as a risk factor. In the subgroup of patients without neurologic symptoms, 9.6 % were diagnosed with neurosyphilis. VDRL titers ≥ 1:32, HIV viral load ≤ 400 copies/mm^3^ and CD4 count ≤ 350 cells/mm^3^ were independent factors for neurosyphilis diagnosis. Therefore, results were consistent among different subgroups and outcome analysis.

The correlation between the increased risk of neurosyphilis in patients with neurological symptoms and higher VDRL titers has been previously described in the literature.^11^^,^^15^, ^16^, ^17^, ^18^ Until 2015, the recommendation to proceed with lumbar puncture was the presence of neurologic symptoms, CD4+ ≤ 350 cells/mm^3^ or VDRL titer ≥1:32.^19^ The recommendation changed in 2015 to only proceed with lumbar puncture in symptomatic patients.^8^^,^^17^^,^^20^ The background for this change was the fact that CSF leukocyte count is commonly elevated in persons with HIV infection during early syphilis^11^ and are of unknown significance in the absence of neurologic signs or symptoms.^12^ In 2018, a retrospective study was published with 59 asymptomatic patients with CD4 < 350 cells/mm^3^ and/or Rapid Plasma Reagin (RPR) titer > 1:32 who were evaluated with lumbar puncture after treated with standard benzathine penicillin G (median 8-months).^21^ Only one patient had neurosyphilis.^21^ In contrast, in our cohort, 15 (11.7 %) of the 128 asymptomatic patients with CD4 ≤ 350 cells/mm^3^ and/or VDRL titers ≥ 1:32 were diagnosed with neurosyphilis. Our findings are in accordance with other recent studies which have shown that CD4+ count and serum VDRL titers, among other factors, were risk factors to neurosyphilis diagnosis in the HIV-infected population.^15^^,^^16^

The correlation between the increased risk of neurosyphilis and the HIV viral load is not appointed by international guidelines.^8^^,^^22^ Previous studies have found that HIV viral load may be a risk factor for neurosyphilis or that use of any highly active antiretroviral therapy before syphilis infection reduced the odds of neurosyphilis.^23^ Although antiretroviral therapy is widely available in our country, patient adherence to therapy is often challenging, especially in more vulnerable populations.^24^ Identifying individuals who are not yet on therapy or who have poor adherence, reflected by detectable viremia in the blood, could help in the diagnosis of possible asymptomatic neurosyphilis cases according to our findings.

This study has some limitations. Some risk factors, such as the presence of neurological symptoms, especially mild symptoms, such as cognitive complaints, may have been under identified due to the retrospective design. Patients did not undergo standardized neurological assessments and were not always evaluated by neurologists, which may have compromised the recording of less apparent neurological symptoms. Likewise, antimicrobial treatment performed may not have been properly. Also, the adherence to the prescribed treatment could not be measured. As the recommendations for performing lumbar puncture in HIV patients changed in 2015, the rate of lumbar puncture performance in asymptomatic patients was significantly lower after that period. Therefore, patients may have been underdiagnosed after this new guideline, however we followed patients in order to observe if neurological symptoms would develop or if they would fail to decrease VDRL titers. Finally, some patients were diagnosed with neurosyphilis based on CSF cytologic criteria only, which can lack specificity, and most of them did not perform a CSF control after treatment. To minimize this potential bias, we excluded patients with other neurologic conditions or alternative diagnosis. We also explored how diagnostic rates would have been impacted if we had used a CSF leucocyte count criteria of ≥ 20 cells/mm^3^ and performed a separate analysis considering only CSF VDRL positive titers as confirmed neurosyphilis criteria with results going in the same direction.

The strengths of this cohort were the relative high number of syphilis cases analyzed in HIV patients, and a long follow-up (2-years). This magnitude reflects the local epidemiology. In Brazil, we have been observing an increasing incidence of syphilis cases, which has reached more than 160,000 cases a year in 2021.^1^ The state of Rio Grande do Sul (Brazil), in which the study was conducted, has the highest prevalence of HIV in the country.^1^^,^^25^ It also belongs to the region with the highest prevalence of acquired syphilis cases, which has shown a 9-fold increase from 2011 to 2021.^1^ Like Brazil, the United States has experienced a rise in syphilis incidence between 2018 and 2022, from 115,000 to more than 207,000 cases.^26^ Therefore, the current epidemiological situation of syphilis is significantly worse than during the periods when the studies informing the changes in diagnostic criteria were conducted. These variations in syphilis prevalence across different locations and time periods affect the pre-test probability of neurosyphilis and may influence study outcomes. Analyses within this high HIV-syphilis prevalence setting are essential to further explore potential risk factors for neurosyphilis beyond the presence of neurological symptoms.

In conclusion, our study showed a correlation between VDRL titers, HIV viral load, CD4 count and neurological symptoms with the diagnosis of neurosyphilis. Incorporating these variables in the decision process of performing lumbar puncture in HIV patients with syphilis may be important, especially in high prevalence settings.

## Funding

None.

## CRediT authorship contribution statement

**Beatriz Arns:** Investigation, Formal analysis, Writing – original draft. **Tarsila Vieceli:** Investigation, Formal analysis, Writing – original draft. **Eduardo Gomes:** Investigation. **Mariana Horn Scherer:** Investigation. **Luisa Nakashima:** Investigation. **Maria Luisa Santos:** Investigation. **Ronara Blos Hepp:** Investigation. **Fernanda Greinert:** Investigation. **Maria Helena Rigatto:** Conceptualization, Supervision, Investigation, Formal analysis, Writing – original draft.

## Conflicts of interest

The authors declare no conflicts of interest.
